# Fatal alcohol intoxication in women: A forensic autopsy study from Slovakia

**DOI:** 10.1186/1471-2458-11-924

**Published:** 2011-12-14

**Authors:** Lubomir Straka, Pavol Zubor, Frantisek Novomesky, Frantisek Stuller, Jozef Krajcovic, Karol Kajo, Jan Danko

**Affiliations:** 1 Institute of Forensic Medicine and Medical Expertises, Jessenius Faculty of Medicine, Comenius University, University Hospital, Kollarova 10, 036 01 Martin, Slovak Republic; 2 Department of Obstetrics and Gynaecology, Jessenius Faculty of Medicine, Comenius University, Martin, Slovak Republic; 3 Department of Pathology, Jessenius Faculty of Medicine, Comenius University, Martin, Slovak Republic

**Keywords:** Alcohol, Intoxication, Women, Autopsy

## Abstract

**Background:**

Plenty of information related to alcoholism can be found in the literature, however, the studies have mostly dealt with the predominance of male alcoholism and data related to addiction in women are desperately scarce and difficult to find. Basic demographic data focusing on the impact of acute alcohol intoxication on the circumstances of death and social behaviour in the alcohol addicted female population are needed especially in the prevention of alcohol related mortality.

**Methods:**

A retrospective forensic autopsy study of all accidental deaths due to alcohol intoxication over a 12-year period was performed in order to evaluate the locations, circumstances, mechanisms and causes of death.

**Results:**

A sample of 171 cases of intoxicated women who died due to blood alcohol concentration (BAC) equal to or higher than 2 g/kg was selected. Among them 36.26% (62/171) of women died due to acute alcohol intoxication (AAI). We noted an increase in the number of deaths in women due to AAI from 2 in 1994 up to 5 in 2005 (an elevation of 150% between the years 1994-2005). The age structure of deaths in women due to BAC and AAI followed the Gaussian distribution with a dominant group of women aged 41-50 years (45.16% and 35.09% respectively). The most frequent place of death (98%) among women intoxicated by alcohol was their own home. The study suggests a close connection between AAI and violence against women.

**Conclusions:**

The increasing number of cases of death of women suffering from AAI has drawn attention to the serious problem of alcoholism in women in the Slovak Republic during the process of integration into "western" lifestyle and culture.

## Background

The forensic and medical practices have confirmed that acute alcohol intoxication (AAI) and deaths connected with it represent one of the most common lethal impacts of substance abuse in any human population. After the accession of Central European countries to the European Union, without exception the influence of drug addiction increased in line with the long-term pertinent lethal impact of alcoholism on society as a whole. Also, the female population is not immune to this phenomenon and we have recorded increasing alcohol consumption among women, particularly young women [1]. In Central European societies, the merits for men and women of alcohol addiction are still very different as a result of the different roles of men and women in the former communist society [2]. One of the fatal consequences of this was a non-selective approach to alcoholism in such a society. Acute feminine alcohol intoxication characteristics cannot actually be specified in the scope of alcoholism of the whole population. This problem has been registered throughout the world. Although the literature deals with alcoholism in families or societies globally [3,4], there is still little information to describe precisely the particularities of alcoholism and the behavioural effects of alcohol in women, and only detailed study may subsequently find solutions to this problem. Basic demographic data focusing on the impact of acute alcohol intoxication (AAI) on the circumstances of death and social behaviour in the alcohol addicted female population are needed especially in the prevention of alcohol related mortality. Thus, using a large cohort design, we aimed to analyse deaths due to accidental alcohol-involved intoxication or "overdoses" in women. Moreover, the presented analysis is the first work in Slovakia monitoring, in detail, the mortality of women in relation to the circumstances of death and the characteristic features of alcohol intoxication.

## Methods

### Study design and setting

A retrospective register-based forensic autopsy study of all deaths of women due to accidental alcohol intoxication (AI) over a 12-year period (1994-2005) was performed in order to evaluate the locations, circumstances, mechanisms and causes of death of women who died due to AAI. Data from the forensic registry of the Office of the Chief Medical Examiner covering the north-west region of the Slovak Republic with 1 million inhabitants were reviewed. All of the forensic and medical investigations, as well as medical expertise, were provided at the Jessenius Faculty of Medicine in Martin, Comenius University of Bratislava. The retrieved forensic data were used for subsequent multi-parametric analysis focusing on the evaluation of circumstances, mechanisms and causes of death in the cases of women who met the basic selection criteria.

### Study population

From 6,131 autopsy reports analysed during the study period, a group of women from the Slovak Republic whose deaths were suspected to be due to AAI was selected (211 cases). By detecting the alcohol concentration in the blood (BAC), we ascertained a value equal to or higher than 2 g/kg in a group of 171 women as the inclusion criterion for the final analysis. The BAC was assessed by gas chromatography [5].

### Ethics and approval

The study design and protocol was approved by the Regional Ethical Committee at the Jessenius Faculty of Medicine registered under number IRB-00005636 at the Office for Human Research Protection, U.S. Department of Health and Human Services. All register connections were performed by a statistical authority and the data were analysed anonymously. The study was conducted in accordance with the guidelines proposed in the Helsinki Declaration.

### Statistics

We used the Student's *t*-test and chi-square test for trends for statistical comparison. A *p *value of less than 0.05 was considered statistically significant. The data were analysed using MedCalc software 11.1. (Mariakerke, Belgium).

## Results

### General descriptive characteristics

During the 12-year period of the study (1994-2005), 171 cases of death in women with a measured alcohol concentration in the blood at the time of death equal to or higher than 2 g/kg were selected. Of these, 62 cases (36.26%) were concluded to be deaths caused by AAI. In all of the cases, the women were of Slovak origin. The BAC and AAI groups did not differ in body mass index, education and ethnicity.

### Distribution of cases over time

**Figure 1:**
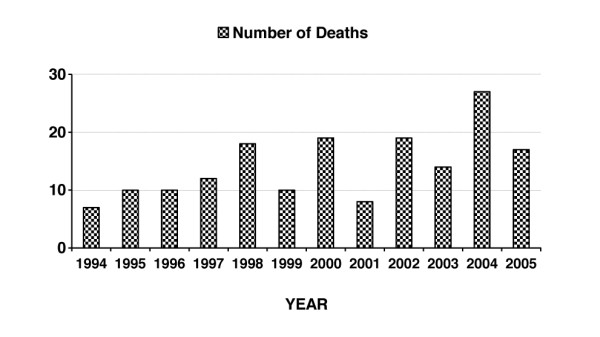
**Number of deaths of woman with blood alcohol concentration equal to or higher than 2 g/kg**.

**Figure 2:**
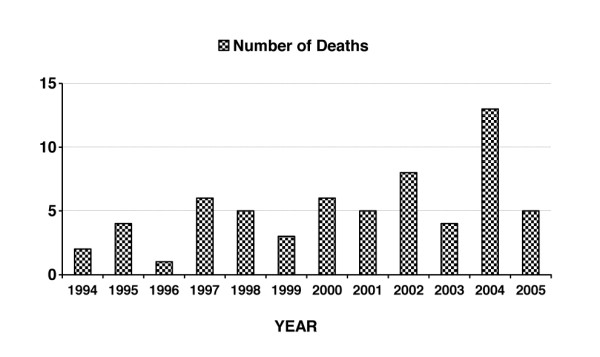
**Number of deaths in subgroup of women with acute alcohol intoxication**.

With regard to cases of death caused by the concentration of alcohol in the blood being equal to or higher than 2 g/kg, the number of such cases during the studied period increased significantly from 7 in 1994 to 17 in 2005 as an endpoint (*p *< 0.01, chi-square 19.83, df = 8), demonstrating a rise of 143% (Figure 1). Simultaneously, the number of women in the subgroup who died due to AAI, rose from 2 in 1994 up to 5 in 2005 as an endpoint (*p *< 0.02, chi-square 20.156, df = 9), representing an increase of 150% (Figure 2).

### Age structure of studied subjects

**Figure 3:**
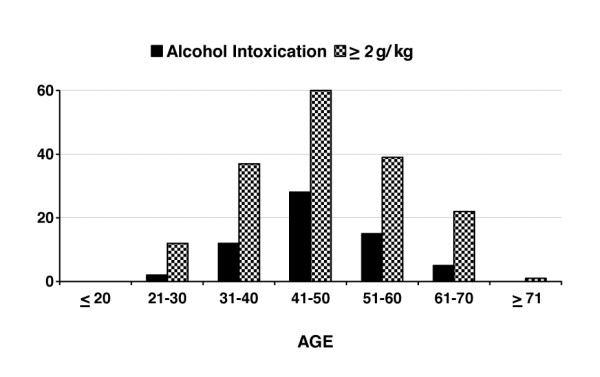
**Age structure of studied subjects**.

Figure 3 shows the age structure of the women who died due to alcohol intoxication. Here the dominant effect of women aged 41-50 years (up to 45.16%) can be seen. The youngest woman who died due to AI was 22 years old (found in the forest); the oldest was 63 (found at home). The average age for the whole analysed BAC group was 47.1 years (12.1 ±SD) and within the alcohol intoxicated subgroup 47.2 years (10.9 ±SD) (*p *= NS, 0.8481, df = 6).

### Place of death

Place-specific mortality analysis showed a dominant proportion of socially hidden excessive amounts of alcohol consumption in the BAC group resulting in an 81% rate of death at home. Second and third were deaths occurring on the roads (9%) and in the countryside (8%). In only 2% of cases, the death was located in different places (e.g. in hospital, on the railway or at social celebratory events). As for the AAI subgroup, 98% of women died at home and only 2% died in other places. The given analysis has shown that alcohol intoxicated women (the BAC group) predominantly die at home, while lethally intoxicated women die almost exclusively in this environment.

### Cause of death

The analysis of the causes of death yielded 8 events directly linked to mortality. The following list shows the highest incidence rates:

The largest group comprises women who died due to direct alcohol intoxication. In this group 36% of women died with a concentration of alcohol in the blood equal to or higher than 2 g/kg.

Surprisingly, 12% of women seriously intoxicated by alcohol were the victims of murder, which means that more than one in ten women with a BAC higher than 2 g/kg has been killed by the hands of a murderer.

Pathological causes of death were determined in 12% of women who died with a BAC equal to or higher than 2 g/kg. Here, the majority (82.67%) of deaths resulted from failure of the cardio-vascular system.

Among the cases of death due to accidents (10%), falls from an upright position to hard ground or falls down stairs occurred most often among registered causes.

Cases of drowning asphyxia were also quite frequent (9%). The most frequent were accidental falls into rivers, natural lakes or swimming pools and water treatment works, when drowning asphyxia was caused by the inability of the deceased for self-rescue due to alcohol intoxication.

Different types of suicides accounted for 8% of cases of death during severe intoxication. In this subgroup, suffocation by hanging was predominant.

Severe hypothermia (8%), followed by acute cardio-respiratory insufficiency, proved to be due to the strong negative vasodynamic effect of ethanol and rapid loss of body temperature, thus enriching the cause-related mortality profile in women with a BAC equal to or higher than 2 g/kg.

**Figure 4:**
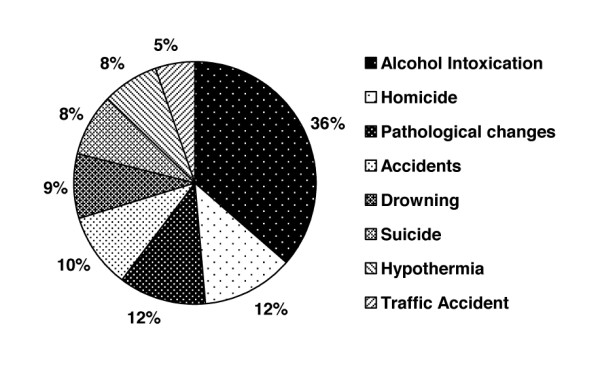
**Cause of death in women with blood alcohol concentration equal to or higher than 2 g/kg**.

Finally, the smallest group of cases of death among seriously intoxicated women represented traffic accidents (5%). In detail see Figure 4.

### Pathological changes

Complete autopsy findings have been analysed in particular for the presence of organ pathology in women who drink. The majority (49%) of women examined post-mortem exhibited liver damage on histopathological analysis. Primarily, it was ethylic steatosis. Alcohol-toxic hepatitis and characteristic micronodular cirrhosis due to alcohol-toxic causes, as reported in other studies from Damjanov and Linder [6] and Rubin and Farber [7], were exclusively rare in our files. Second, alcoholic cardiomyopathy was found in 35% of cases, mostly in the form of myelofibrosis. Furthermore, various anomalies of cardiomyocytes were also found in the macroscopic view of concentric cardiac hypertrophy. In 22% of deaths of women with serious alcohol intoxication, pathological damage to the respiratory system was also found, most often originating from chronic bronchitis. The increased presence of gynaecological malignant diseases was not registered.

## Discussion

Female alcoholism in Slovakia still remains a taboo topic at the beginning of the 21st century. Partially, this is due to the long-term isolation of the Slovak Republic in the former Eastern Block, this country is still not significantly affected by drugs--more so by alcohol. It was, and still remains, the most frequent lethal psychotropic substance. Current society is still more likely to accept alcohol consumption among males rather than females. [2]. Because of this "positive segregation" there is little information about female alcoholics available among experts. This presented analysis of cases of the deaths of women related to excessive alcohol abuse is the first of its kind and brings a substantially different view of the issue of excessive alcohol abuse by women in Slovakia and shows a number of interesting specifics of acute female alcoholism (ethylism). Moreover, the study impact is increased by the fact that only a few studies exist in the literature which evaluate the behavioural effects of alcohol in women and the factors that may influence their response to alcohol intoxication. However, none of them focused on alcohol intoxication-related mortality.

Across the regions studied, the number of female deaths involving alcohol intoxication was equivalent to just 5% of the number of deaths due to cervical cancer [8]. Even though in this context the number appears low, it should be kept in mind that in the case of alcoholic intoxication, these deaths were not caused by a pathological change, but instead, solely by the failure of an individual to exercise self-restraint. Alcohol consumption among women is increasing, particularly in young women [1] and this group is showing a higher rate of a lifetime prevalence of frequent drinking compared to the older cohort of women. In our sample, as many as 98% of women in the state of alcoholic intoxication died at home. It appears, therefore, that the aforementioned type of hidden alcohol abuse is dominant among women. Like other studies [9], our analysis also indicates a predominance of such deaths in women in their fifties.

The analysis also shows that if women reach a 2 g/kg or higher concentration of alcohol in their blood, more than one third of them tend to continue drinking until the lethal end. This is probably related to the considerably higher sensitivity of the female organism to the toxic effects of ethanol and its metabolites [10]. Moreover, lower body water content in females compared with males results in the dispersal of an identical quantity of consumed alcohol in a smaller quantity of water, as a result of which a woman will reach higher ethanol concentration. Similarly, the lower activity of enzymes that decompose alcohol can also help to explain the increased vulnerability of the female organism to alcohol. Another assumption that should be mentioned in this context is that hormonal changes in the course of the menstrual cycle are likely to affect the variable toxic sensitivity of female organisms [11]. Furthermore, it was proved that alcohol consumption enhances some dysphoric moods, anxiety or depression during the luteal phase of menstrual cycle [12], probably due to premenstrual symptoms. Finally, when considering alcohol consumption among women, one cannot omit the modifying factors such as genetic predisposition, environmental circumstances and social status (e.g. homelessness, low-income) which influence the development of drinking patterns [13,14] or different behavioural and psychological acceptances or perceptions of external inputs resulting in chronic psychiatric problems, known to be more penetrative among women. The last factor that has been shown to alter the response to alcohol in women is a family history of alcoholism, which is one of the greatest predictors of subsequent alcohol abuse [15]. Obviously, the variable spectra of factors may have an impact on the alcohol-dependent behaviour in women.

It is a known fact of forensic practice that as many as 50% of all road accidents are caused by a person under the influence of alcohol [16], an item of data comparable to our experience. In the case of women, their numeric representation among road accident victims is markedly lower, which is potentially due to the continuing lack of social acceptance of women visiting public facilities that serve alcohol (the stigma of women drinking in public) and thus women are "spared" various road accidents.

The association of alcohol abuse and suicide is considered to be relatively frequent due to the

psychological "booster effect" in the case of female depression [17] and the alcohol-induced depression of males [18]. Comstock [19] and Scolan [20] refer to as many as one quarter of all suicide victims being under the influence of alcohol. In our sample the group of suicides of heavily intoxicated women was numerically rather similar to the male suicide victims (8%). In our study, a socially significant and unexpectedly large group was the one comprising victims of violence who were heavily intoxicated when they were killed by another person. The result of the analysis is as follows: one tenth of women who died with alcohol concentration in the blood equal to or higher than 2 g/kg died as a result of an aggressive attack by a third person, which is also interesting from the point of view of victimology [21,22]. The link between alcohol intoxication and violence has also been documented in similar studies from the USA and Finland [23,24].

In the group of women who died of pathological causes while being heavily intoxicated, the most frequent occurrence was alcoholic cardiomyopathy, especially dilated cardiomyopathy, with apparent malnutritional, metabolic and toxic causes [6,25]. In contrast, alcohol-toxic hepatitis and characteristic micronodular cirrhosis caused by alcohol-toxicity [6,7] were virtually rare in our sample, which is a fact that we have not been able to explain so far. The third characteristic pathological change was the occurrence of chronic, usually mucopurulent inflammation of the respiratory tract, which is a typical symptom of alcoholics [26].

We observed the highest incidence of AAI and alcohol related deaths in 2004. It is quite difficult to find the exact underlying reasons for this. However, we suggest several factors as triggers, acting together leading to such a rise in BAC and AAI deaths. First, there was an immense decrease in social status for the whole population in 2004 due to the economic requirements dictated by the Maastricht Treaty prior to Slovakia entering the European Union. Thus, social support from public state sources was highly restricted. Second, the reason could also be "open" access to the western lifestyle which has become much more affordable for people following Slovakia's entry into the European Union in 2005. Finally, the UDZS (Health Care Surveillance Authority) was established in 2004 in Slovakia. The institute controls health care quality and the activities of health care providers, thus many general practioners attending the deceased had increased the number of autopsies for fear of the consequences of un-autopsied forensic deaths.

## Conclusion

The status of women in society today has undergone significant changes and women have greater economic and moral freedom. It is difficult to assess whether the increase in drinking in women is the price that women pay for their emancipation. It is customary and understandable that, in the case of death due to alcohol poisoning (intoxication), close family members or neighbours try to play down and cover up this fact, especially if the deceased person was a woman. This approach has been subconsciously accepted by society as a whole, which is the main reason why the evaluation of specific features of female excessive alcohol abuse is impeded by there being less information available about the circumstances of such deaths. Nevertheless, on the basis of the above analysis, we can conclude that female alcoholism has its specific features, which we can currently identify and name. The distinct rise in the trend of women dying in the state of AAI in the studied region of Slovakia stopped in 2005, which could relate to the economic stabilization of the population of a post-communist country at the time of EU accession. However, this fact should be viewed very carefully as it is possible that the data from 2005 may be just a dip within a trend as no further years were analysed. Of all female deaths caused by alcohol intoxication, 98% happened at home; caused by the pervasive prevailing trend of social non-acceptance of women drinking in public. Even though there is no doubt that the current post-modern and "post-moral" society and the related feminization will at some point break this taboo as well, the submitted paper has documented the continuing cultural phenomenon of domestic alcoholism. The issue of female alcoholism is still in the "grey zone", behind closed doors. This study has singled out a group of 41-50-year old women as being at the greatest risk of AAI with lethal consequences. However, the small number of cases and lack of population data in our study are the limitations of conclusions and warrants about inappropriate generalizations in certain populations. Among women who died with a concentration of alcohol in the blood equal to or higher than 2 g/kg, as many as 12% died as a result of violence committed against them, which suggests the correlation of excessive alcohol abuse and violence against women. Therefore, based on the retrieved data from our study, which represents one of the larger studies to date evaluating alcohol-dependent women's mortality, we can conclude that precise knowledge of all detailed specifics of female excessive alcohol consumption is an important step in the endeavour to reverse the rising trend of alcohol intoxication-related mortality in women worldwide.

## Competing interests

The authors declare that they have no competing interests.

## Authors' contributions

LS, FS and JK provided the medical and clinical expertise and support. LS is the principal investigator who designed the study, wrote the protocol and majority of the paper. PZ contributed to the conceptualization of the analysis and paper, he wrote and reviewed drafts, provided statistical and graphical analysis, and performed clinical searches in case histories. KK was responsible for the histopathological analyses and he managed the literature search and summaries of previous related works. FN and JD reviewed drafts of the paper and coordinated the study implementation. All authors contributed to and have approved the final manuscript.

## Pre-publication history

The pre-publication history for this paper can be accessed here:

http://www.biomedcentral.com/1471-2458/11/924/prepub
